# Extracellular Vesicles Generated by Mesenchymal Stem Cells in Stirred Suspension Bioreactors Promote Angiogenesis in Human-Brain-Derived Endothelial Cells

**DOI:** 10.3390/ijms25105219

**Published:** 2024-05-10

**Authors:** Jolene Phelps, David A. Hart, Alim P. Mitha, Neil A. Duncan, Arindom Sen

**Affiliations:** 1 Pharmaceutical Production Research Facility, Schulich School of Engineering, University of Calgary, 2500 University Drive N.W., Calgary, AB T2N 1N4, Canada; jolene.phelps@ucalgary.ca; 2 Department of Biomedical Engineering, Schulich School of Engineering, University of Calgary, 2500 University Drive N.W., Calgary, AB T2N 1N4, Canada; hartd@ucalgary.ca (D.A.H.); amitha@ucalgary.ca (A.P.M.); 3 McCaig Institute for Bone and Joint Health, Cumming School of Medicine, University of Calgary, 3280 Hospital Drive N.W., Calgary, AB T2N 4Z6, Canada; duncan@ucalgary.ca; 4 Department of Surgery, Cumming School of Medicine, University of Calgary, 3330 Hospital Drive N.W., Calgary, AB T2N 4N1, Canada; 5 Faculty of Kinesiology, University of Calgary, 2500 University Drive N.W., Calgary, AB T2N 1N4, Canada; 6 Department of Clinical Neurosciences, Cumming School of Medicine, University of Calgary, 1403 29 Street N.W., Calgary, AB T2N 2T9, Canada; 7 Department of Civil Engineering, Schulich School of Engineering, University of Calgary, 2500 University Drive N.W., Calgary, AB T2N 1N4, Canada; 8 Department of Chemical and Petroleum Engineering, Schulich School of Engineering, University of Calgary, 2500 University Drive N.W., Calgary, AB T2N 1N4, Canada

**Keywords:** extracellular vesicles, angiogenesis, mesenchymal stem cells, cerebral microvascular endothelial cells, ischemia, physioxia, bioprocessing, stirred suspension bioreactors

## Abstract

Interrupted blood flow in the brain due to ischemic injuries such as ischemic stroke or traumatic brain injury results in irreversible brain damage, leading to cognitive impairment associated with inflammation, disruption of the blood–brain barrier (BBB), and cell death. Since the BBB only allows entry to a small class of drugs, many drugs used to treat ischemia in other tissues have failed in brain-related disorders. The administration of mesenchymal stem cell (MSC)-derived extracellular vesicles (EVs) has shown promise in improving the functional recovery of the brain following cerebral ischemia by inducing blood vessel formation. To facilitate such a treatment approach, it is necessary to develop bioprocesses that can produce therapeutically relevant MSC-EVs in a reproducible and scalable manner. This study evaluated the feasibility of using stirred suspension bioreactors (SSBs) to scale-up the serum-free production of pro-angiogenic MSC-EVs under clinically relevant physioxic conditions. It was found that MSCs grown in SSBs generated EVs that stimulated angiogenesis in cerebral microvascular endothelial cells, supporting the use of SSBs to produce MSC-EVs for application in cerebral ischemia. These properties were impaired at higher cell confluency, outlining the importance of considering the time of harvest when developing bioprocesses to manufacture EV populations.

## 1. Introduction

The high rate of oxidative metabolism imposed by brain activity accounts for ~20% of all available oxygen utilized in the body [1], and tight regulation of blood flow and oxygen delivery in the brain is critical to survival. When this blood flow is interrupted, such as during an ischemic stroke or following a traumatic brain injury (TBI), the resulting lack of oxygen, termed cerebral ischemia, causes metabolic alterations that can result in irreversible brain damage, subsequently leading to cognitive impairment associated with abnormal mitochondrial activity, inflammation, disruption of the blood–brain barrier (BBB), and ultimately, cell death [2]. The process of angiogenesis in the brain, where new vessel formation replenishes the blood supply to an infarcted area, has been shown to be critical for recovery following cerebral ischemia, and also to contribute to neurogenesis and improved neurological function [3,4]. A challenge in drug development for brain-related disorders is that the BBB only allows a small class of drugs (i.e., those that exhibit both high solubility and low molecular weight) to cross into the brain, preventing the transport of 98% of small-molecule drugs and 100% of large-molecule drugs [5]. 

Recently, mesenchymal stem cell (MSC)-derived extracellular vesicles (EVs) have shown promise for inducing angiogenesis and improving functional recovery in the brain [6,7,8]. EVs are lipid-membrane-bound nanoparticles (30–200 nm) that contain an abundance of bioactive proteins, lipids, and RNAs, and are produced in large numbers by cells. EVs are gaining clinical interest due to their inherent delivery and homing mechanisms and their non-living nature, which enables them to be stored and transported more easily relative to cells. MSC-EVs are of special interest for the treatment of cerebral conditions due to their ability to cross the BBB and their lack of immunogenicity [6,9]. Phase I clinical trials have demonstrated the safety of administering allogeneic MSC-EVs [10,11], and pre-clinical trials have demonstrated their ability to incorporate into neurons and the microglia of the forebrain when inhaled intranasally [12,13]. When administered following ischemic stroke and TBI, they have been shown to exhibit promising therapeutic benefits, including the induction of angiogenesis and neurogenesis, improved neurological recovery, and reduced inflammation via the manipulation of endogenous neural precursors and endothelial cells [14,15,16,17].

MSCs are highly sensitive to their culture environment; thus, the conditions and platform in which they are grown influences their capacity for growth, their therapeutic properties, and, in turn, the therapeutic properties of the EVs they secrete [18]. Previously, we showed that MSC-EVs generated in static tissue culture flasks (T-flasks)-enhanced angiogenesis of cerebral microvascular endothelial cells (CMECs), and these effects could be further enhanced by conditioning the MSCs under physioxic conditions (i.e., a low oxygen environment that mimics physiological oxygen exposure) [19]. However, T-flasks are not amendable for scaling-up production of MSCs or their corresponding secreted products due to the low surface area available for cell growth. Obtaining clinical quantities would require the utilization of a large number of T-flasks, which would be manually intensive, increase the risk of contamination, and introduce variability into the manufacturing process [20]. Bioreactors have been shown to be an efficient scalable platform for the generation of MSC populations [20,21,22] and, more recently, scalable MSC-EV production has been demonstrated in hollow fiber bioreactors [23,24,25], vertical wheel bioreactors [26], stirred-tank or suspension bioreactors (SSBs) [27,28], perfusion bioreactors [29], and flat plate bioreactors [30], which have reported higher EV production rates per cell, leading to greater EV concentrations and increased yields relative to static culture. Mechanical stimuli, such as fluid shear stress present in SSBs, have been shown to influence MSC behavior and their EVs [18]. Fluid shear stress has been demonstrated to enhance MSC-EV production [26,31], with EVs isolated from cultures exposed to shear stress exhibiting similar therapeutic benefits to those isolated from static culture [28,30,31].

In the current study, we evaluated the feasibility of using SSBs to scale-up the production of MSC-EVs and compared the angiogenic capacity of these EVs to those generated in traditional static T-flasks, both under clinically relevant physioxic (3% O_2_ in the headspace), serum-free conditions. We further evaluated whether the time of harvest from the SSBs could influence the composition and functionality of isolated EV populations. As MSCs are highly influenced by their environment, understanding how shear stress in a dynamic culture affects the properties of the EV populations they produce is crucial to the development of bioprocesses aimed at manufacturing clinical quantities of functional EVs to treat brain-related disorders.

## 2. Results 

### 2.1. SSB Culture Affects MSC Proliferation and Gene Expression, with Dependence on Confluence

**Figure 1:**
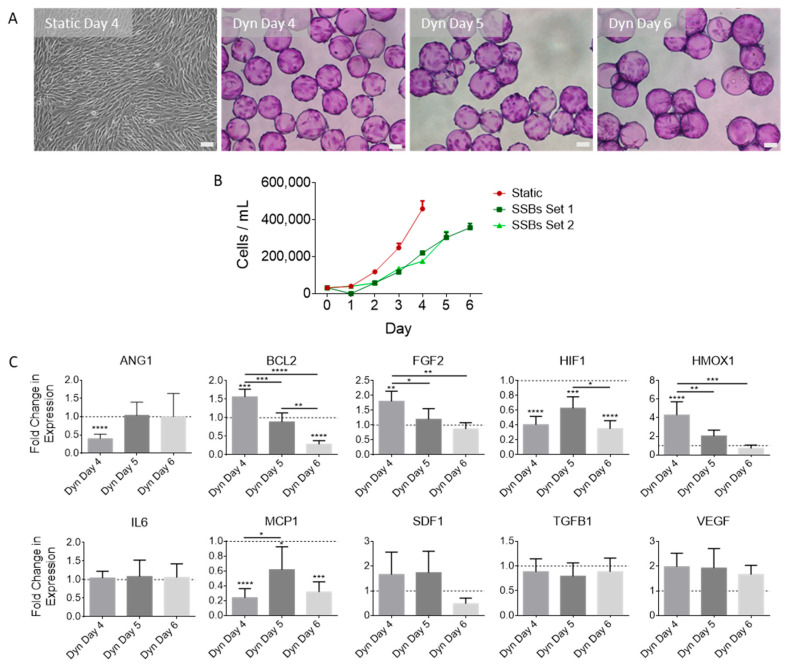
(**A**) Photomicrographs of MSCs on day 4 post-inoculation in static conditions in T-75 flasks, and on days 4, 5, and 6 under dynamic conditions in SSBs (Dyn) on microcarriers. Microcarrier samples were fixed and stained with crystal violet for imaging (scale bars represent 100 µm). (**B**) Cell density was measured every 24 h (N = 3, error bars represent standard deviation). SSBs set 1: growth medium replaced on day 2 or day 4 with EV collection medium (EV harvest day 4 or 6 respectively). SSBs set 2: growth medium replaced on day 3 with EV collection medium (EV harvest day 5). (**C**) Fold change in MSC gene expression from SSB conditions relative to day 4 static controls (N = 5, errors bars represent standard deviation). Stars above columns indicate statistical significance relative to static conditions: * *p* < 0.05, ** *p* < 0.01, *** *p* < 0.001, **** *p* < 0.0001. Abbreviations: ANG1, angiopoetin-1; BCL2, B-cell lymphoma 2; FGF2, basic fibroblast growth factor; HIF1, hypoxia inducible factor 1 subunit alpha; HMOX1, heme oxygenase 1; IL6, interleukin 6; MCP1, monocyte chemoattractant protein 1; SDF1, stromal cell-derived factor 1; TGFB1, transforming growth factor beta 1; VEGF, vascular endothelial growth factor A.

MSC populations were expanded in a serum-free, physioxic (3% O_2_) environment under either static conditions in T-flasks or dynamic conditions in 100 mL SSBs. It was observed that the rate of cell proliferation in T-flasks was significantly higher than that observed in SSBs. The MSCs in T-flasks were harvested on day 4, whereas those grown in SSBs were harvested on days 4, 5, or 6 (visualized in Figure 1A). To maintain a constant post-inoculation time frame, MSCs harvested from static culture on day 4 were compared to MSCs harvested on day 4 in SSBs. However, the cells in SSBs were also evaluated on days 5 and 6 as the cell density under dynamic conditions increased towards that observed on day 4 in static culture. Samples were taken daily to evaluate changes in growth (Figure 1B). RT-qPCR was performed on the cells at the time of EV harvest. Figure 1C compares the fold change in expression of MSCs grown in SSBs and harvested on days 4, 5, and 6 normalized to MSCs grown in static T-flasks. Day-4 gene expression analysis of MSCs cultured in dynamic SSBs and static T-flasks revealed reduced ANG1, HIF1, and MCP1 expression and increased BCL2, FGF2, and HMOX1 expression in cells from SSBs. A non-significant increase in VEGF and SDF1 expression was found in SSB cultured cells, while TGFB1 and IL6 levels remained stable. Comparing MSCs harvested at different times (increasing in cell confluence) in SSBs, a reduction in the expression of BCL2, FGF2, and HMOX1 was found for cells at higher confluency (i.e., highest expression at day 4, decreasing to day 6).

### 2.2. Dynamic Culture Conditions and Cell Confluency Alter EV Secretion and Composition

**Figure 2:**
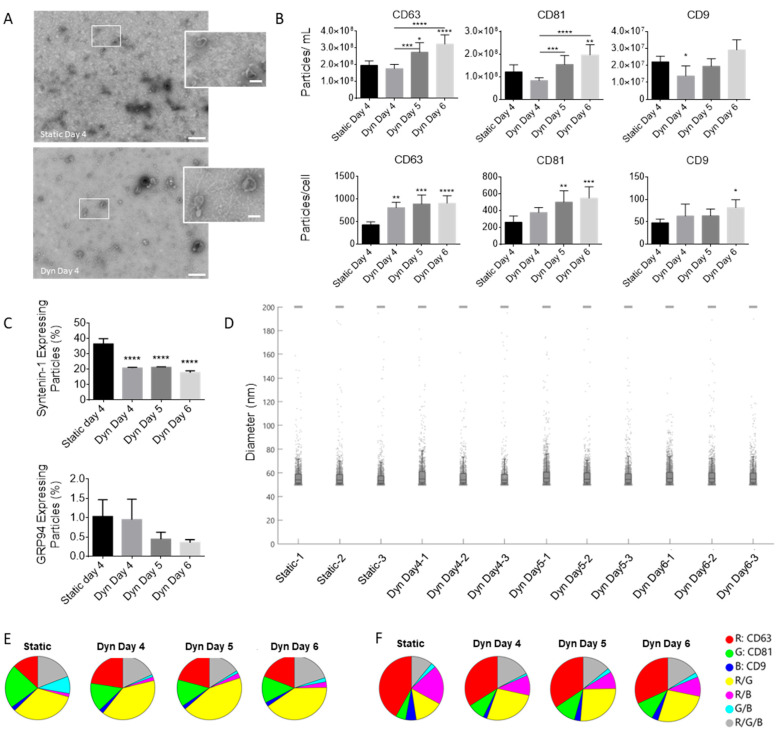
(**A**) Representative TEM image, with the boxed section zoomed in below (scale bar = 250 nm; zoomed in scale bar = 125 nm). Typical cup-shape morphology of EVs is observed. (**B**) CD63/81/9 total particle counts for static and SSB (Dyn) conditions as measured by SP-IRIS analysis (N = 3). (**C**) Percentage of syntenin-1 and GRP94 expressing particles as a ratio of total CD63 expressing particles (N = 3). (**D**) Sizing data from SP-IRIS analysis (N = 3). (**E**,**F**) Co-localization charts for representative EV samples bound to CD63 and CD81, respectively, from each condition. Error bars represent standard deviation. Stars above columns indicate statistical significance relative to static conditions: * *p* < 0.05, ** *p* < 0.01, *** *p* < 0.001, **** *p* < 0.0001.

**Figure 3:**
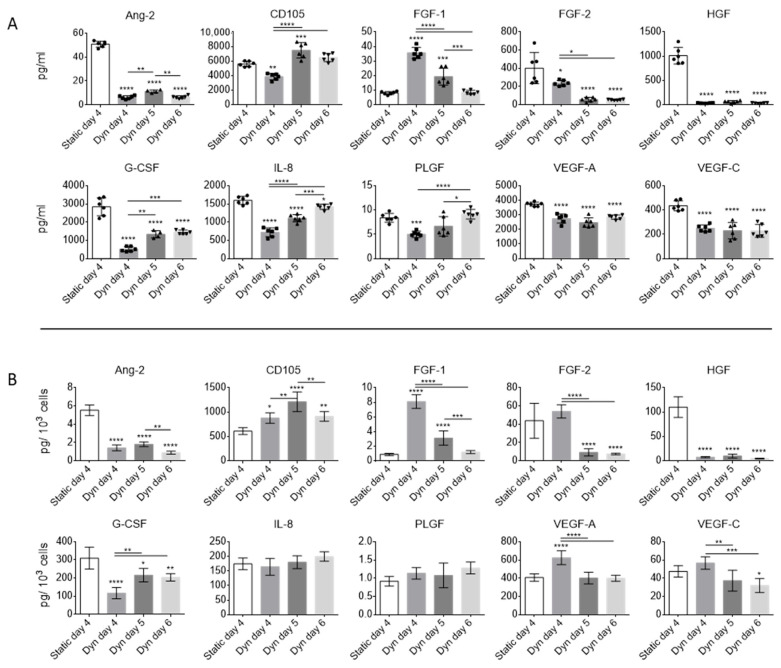
Angiogenic protein concentrations (as measured by Luminex) in EV fractions obtained from MSCs cultured under static and SSB (Dyn) conditions. (**A**) Total protein concentration (pg/mL). (**B**) Protein yield (pg per 10^3^ cells). EV samples are concentrated 40× compared to CM. Error bars represent standard deviation. Stars above columns indicate statistical significance relative to static conditions: * *p* < 0.05, ** *p* < 0.01, *** *p* < 0.001, **** *p* < 0.0001. Abbreviations: Ang-2, angiopoetin-2; CD105, endoglin; FGF-1, acidic fibroblast growth factor; FGF-2, basic fibroblast growth factor; G-CSF, granulocyte colony stimulating factor; HGF, hepatocyte growth factor; IL-8, interleukin-8; PLGF, placental growth factor; VEGF-A/C, vascular endothelial growth factor A/C.

EVs were isolated from the expended medium at time of harvest for each condition via differential ultracentrifugation. The EVs were subjected to TEM imaging to confirm EV morphology and size (Figure 2A), SP-IRIS analysis using an ExoView device to compare particle concentrations expressing EV specific markers, size, and phenotype (Figure 2B–F), and Luminex multiplex analysis to determine angiogenic protein concentrations (Figure 3). Total EV concentration (particles/mL) in SSBs increased in a time-dependent manner and was highest on day 6. Despite the lower cell concentration in SSBs, the EV yield (particles/cell) was significantly higher for all SSB conditions compared to static (based on CD63 positive particles). No significant differences were found for EV yields between SSBs harvested on different days, suggesting that EV production rates in SSBs are not impacted by MSC confluence or time in culture between days 4 and 6. The percentage of syntenin-1-expressing particles was significantly higher in static conditions compared to SSBs, indicating a change in the overall ratio of exosomes to microvesicles, as sytenin-1 is recognized as a marker specific for exosomes [32]. The percentage of GRP94-expressing particles (contaminating cellular debris) was <1% for all conditions, indicating the efficacy of the isolation process that was utilized. No significant differences were found in the average size of EVs in the populations isolated from the different conditions. Figure 2E,F visualize the co-localization of EV-specific markers on the particles bound to CD63 (E) and CD81 (F) capture probes and show minimal changes to the phenotype of the EVs from static and SSB conditions.

The overall concentrations of angiogenic proteins Ang-2, FGF-2, HGF, G-CSF, IL-8, PLGF, VEGF-A, and VEGF-C within the EV fraction were reduced under dynamic conditions (Figure 3A). FGF-1 was higher in dynamic conditions on days 4 and 5, but similar to static on day 6. CD-105 was highest on day 5 under dynamic conditions. On a per-cell basis (Figure 3B), Ang-2, G-CSF, and HGF concentrations were consistently lower in EV fractions obtained from dynamic conditions. VEGF-A and FGF-2 yields were highest in dynamic conditions on day 4 but were reduced at higher confluence. CD-105 yield was consistently higher in dynamic conditions and highest on day 5. The reduced yield of CD-105 on day 6 as compared to particle concentrations seen previously in Figure 2B could indicate that the expression of CD-105 was altered at high confluence. IL-8 and PLGF had stable yields, indicating that dynamic culture did not affect their secretion. 

### 2.3. EVs Isolated from MSCs Cultured in SSBs Have Improved Angiogenic Properties

**Figure 4:**
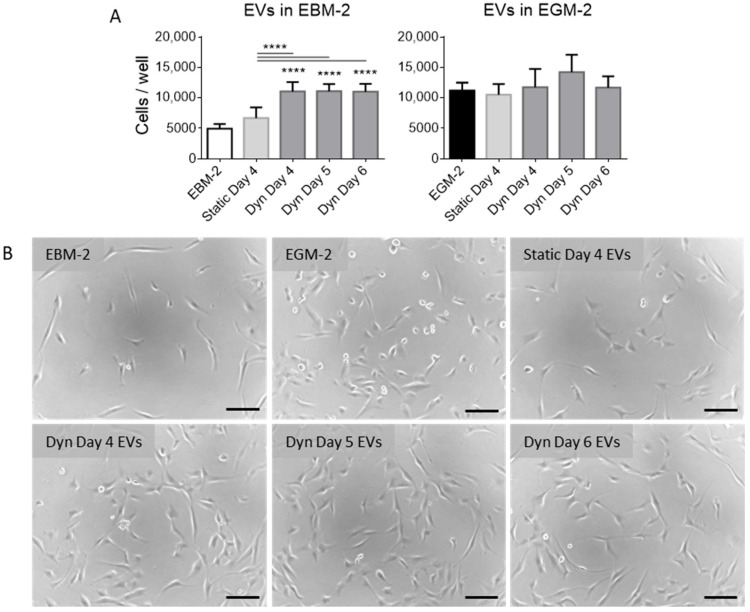
(**A**) CMECs/well after 48 h of culture with/without MSC-EV fractions as measured by DNA content (N = 6). CMECs were inoculated at 5000 cells/cm^2^ in 96-well plates, and EVs were added after 12 h of culture in EGM-2. EVs were resuspended in either EBM-2 or EGM-2 for comparison. Columns represent mean and standard deviation. Stars above columns represent significant differences as compared to the control of EBM-2 with no EVs added: **** *p* < 0.0001. (**B**) Photomicrographs of CMECs 48 h following treatments with EVs resuspended in EBM-2. Scale bar = 100 µm.

EV fractions were isolated from MSCs cultured in static and SSBs and added to cultures of CMECs to evaluate their stimulatory effect on CMEC proliferation and tube formation. EVs were resuspended in endothelial basal medium (EBM-2) to evaluate their stimulatory effect in the absence of supplements, and in endothelial growth medium (EGM-2) to evaluate if they could augment CMEC proliferation in the presence of supplements. It was found that EVs isolated from MSCs cultured in SSBs and resuspended in EBM-2 stimulated proliferation in CMECs significantly more than the control (EBM-2 without supplemented EVs) and those isolated from static culture (Figure 4). As static and dynamic day 4 fractions contained an equivalent number of EVs, and day 5 and 6 fractions from SSBs contained a significantly higher number of EVs, the potency of these EV fractions does not appear to be related to EV quantity. EVs isolated from static MSCs had no significant effect on proliferation. EVs resuspended in EGM-2 had no significant effect on CMEC proliferation (visualized in Supplementary Figure S1).

**Figure 5:**
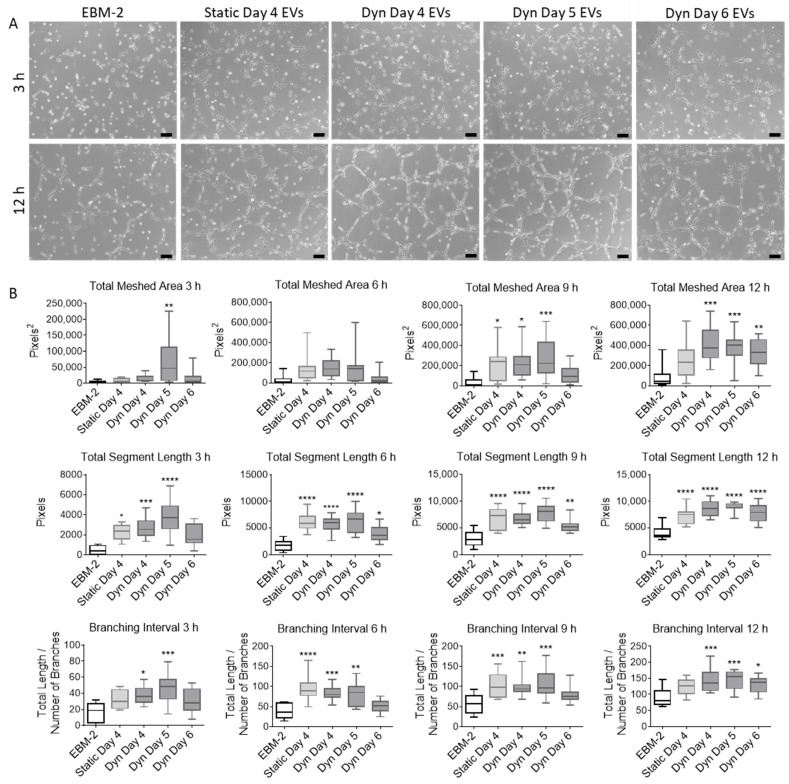
(**A**) Phase contrast images of CMEC tubes formed after 3 and 12 h on a Geltrex matrix exposed to EBM-2 (control), or EBM-2 with the addition of EVs isolated from static MSC cultures on day 4 or SSB (Dyn) MSC culture on days 4, 5, or 6 (scale bar = 100 µm). Images are only shown for 3 and 12 h as these show the most-pronounced differences between the conditions. (**B**) Quantification of CMEC tube formation at 3, 6, 9, and 12 h as measured by ImageJ using the Angiogenesis Analyzer. Error bars represent standard deviation. Stars above columns indicate statistical significance relative to control EBM-2 with no EVs added: * *p* < 0.05, ** *p* < 0.01, *** *p* < 0.001, **** *p* < 0.0001.

EVs produced under static and dynamic SSB conditions were resuspended in EBM-2 and added to CMECs plated on Geltrex coated wells. Tube formation was evaluated at 3, 6, 9, and 12 h. EVs from all conditions were found to have significant stimulatory effects on CMEC tube formation (Figure 5). Total meshed area (area encompassed by fully closed tube networks), total segment length (total length of all tube segments), and branching interval (mean length between two branching segments) were evaluated using ImageJ Angiogenesis Analyzer. EVs harvested from SSBs on day 5 resulted in the highest CMEC meshed area, segment length, and branching interval at 3 h, suggesting that these EVs may have had the most potent effects. EVs from SSBs on day 6 appeared to have lower stimulatory function compared to the other EV populations, despite the larger number of EVs as measured by CD63, CD81, and CD9 concentrations (Figure 2B).

## 3. Discussion

With numerous studies demonstrating the benefits of EVs for the treatment of neurological disorders, there is a need to develop robust bioprocesses that can produce large amounts of therapeutically relevant EVs in a reproducible and scalable manner. This study demonstrated that MSCs-EVs could be produced in SSBs under physioxic (3% O_2_) conditions in a non-proprietary serum-free medium, and that these EV populations are just as effective in stimulating angiogenesis in CMECs as compared to MSC-EVs produced in static T-flasks. This study further demonstrated that EVs harvested from SSBs on day 6 (i.e., high confluence) may be less effective in inducing CMEC tube formation, which outlines the importance of harvesting MSCs and their EVs at earlier time points in applications of angiogenesis. 

Similar results were seen in recent studies by Costa et al. [27] and Kronstadt et al. [29], both demonstrating increased EV production in bioreactor culture (in SSBs and perfusion bioreactors respectively), as well as the preservation of angiogenic bioactivity in HUVEC tube formation and migration assays from EVs generated under both culture modes. Differences do exist between the methods used in the current study. Costa and Kronstadt compared EV production in the two modes (static vs. SSB or perfusion) optimized for MSC growth—initial inoculation density and surface area differed significantly between the two cultures being compared. In addition, the basis for comparison in HUVEC assays was the number of EVs, quantified by nanoparticle tracking analysis (NTA). The current study compared EV production between static and SSBs when cells were inoculated at the same inoculation density and surface area/volume ratio to maintain process inputs. It was reassuring that once the processes were optimized for MSC growth, similar results were seen in terms of EV production. To account for differences in cell confluence between the two conditions, the current study evaluated three time points in SSBs. All conditions were compared in CMECs to evaluate EV functionality as it relates to angiogenesis within the brain. Conditions were compared on a basis of a CM volume, as opposed to an EV quantity, for two reasons: (i) there is no method currently available that can accurately quantify EVs (i.e., NTA counts non-EV particles such as protein aggregates and lipoproteins, and SP-IRIS selects for EVs expressing only CD63, CD9 or CD81); (ii) the aim of this study was to compare specific processes; therefore, all comparisons were conducted on the basis of equal process inputs on day 0. Both methods present their own advantages and disadvantages.

The current study used PPRF-msc6 medium throughout the cell culture process. PPRF-msc6 is a clinically applicable, chemically defined serum-free culture medium developed specifically for the culture of MSCs [33]. The use of serum-free medium throughout the process was important as serum can contain infectious agents, and significant batch-to-batch variability exists, which can hinder process reproducibility [34]. Serum-free media are essential in EV production processes due to the presence of EVs in serum. An important consideration when using serum-free culture media is the higher abundance of proteins, such as albumin and fetuin, which can co-isolate with the desired EV fractions. All EV collection media used in the current study were ultracentrifuged overnight to discard pelleted contaminating proteins. MSC proliferation and gene expression were studied and compared between static and SSBs (as EV populations are reflective of the state of their parent cells). MSCs exhibited lower proliferation rates in SSBs compared to static T-flasks. The primary differences between the two culture platforms were that SSBs introduced fluid shear (1.6 dyn/cm^2^), supported higher nutrient and oxygen transfer, and involved attachment and growth on the convex surface of microcarrier beads. Luo et al. demonstrated that consistent laminar shear stress reduced MSC proliferation, with cells arrested in the G_0_ or G_1_ phase, and suppressed apoptosis via increased expression of BCL2 [35]. While other studies have reported increased MSC proliferation with shear stress [36,37], the conditions of these previous studies differed in that they were exposed to static conditions intermittently or following shear exposure. In addition, it has been reported that only high levels of oscillatory shear (20 dyn/cm^2^) can induce changes in proliferation [37]. Cellular contractility has been shown to be increased by shear flow [38] and by culture on a convex surface [39] and could further explain the reduction in proliferation rate, as intracellular contractility reduces cellular proliferation [40,41] and increases adipogenesis of MSCs [42]. It should be noted that despite the achieved cell density in the current study being lower in SSBs, the scalability of this platform can result in a much greater quantity of cells and therefore EVs, produced in a single vessel under very controlled conditions relative to static culture vessels.

The changes in gene expression following exposure to shear stresses in SSB culture are consistent with the literature and can be compared between day 4 cultures in static and SSBs. Ang-1 is a critical player in vessel maturation and is involved in mediating the migration, adhesion, and survival of endothelial cells (ECs) [43]. Tie1, known to enhance Ang-1 signaling, was previously shown to be downregulated in endothelial cells exposed to shear stress [44], which could explain the mechanism by which Ang-1 was downregulated. Longer exposure to shear stress was found to normalize Ang-1 expression levels [44], consistent with the present study. Physiologically, the downregulation of Tie1 and Ang-1 may be required for the destabilization of ECs to initiate the process of vascular restructuring [44]. However, Ang-1 is a strong inducer of EC sprouting and reduces EC permeability; therefore, longer exposure times to shear (i.e., EVs collected on day 5 as opposed to day 4 in the current study) may be of benefit in generating functional MSC/MSC-EV populations [45]. Intermittent exposure of MSCs to shear stress has demonstrated upregulation of the angiogenic genes VEGF and FGF2 and downregulation of HIF1 [46], consistent with our findings. Shear stress increases the expression of endothelial cell markers and promotes the endothelial differentiation of MSCs, upregulating the production of angiogenic growth factors [47,48,49]. The downregulation of HIF1 is representative of more efficient oxygen transport. MCP-1 expression correlated with that of HIF1, as previously shown in astrocytes [50].

Similarly, changes in gene expression with increasing confluence can be compared between SSB conditions. Increasing cell culture density has previously been found to reduce the expression of genes related to the maintenance of stem cell qualities, as well as those associated with the cell cycle, cell senescence, and regulation of the actin cytoskeleton [51,52]. Downregulation of FGF2 has been reported to slow proliferation due to the attainment of confluence [51]. In addition to FGF2, we also observed a consistent downregulation of BCL2 and HMOX1 with increasing confluence, likely due to similar mechanisms, as HMOX1 inhibition has been shown to reduce proliferation, migration, adhesion, and clone formation processes of MSCs [53]. HMOX1 is a downstream target of HIF1 and is associated with protection against ischemic injury [54] and enhanced VEGF synthesis [55]. The stability of IL6 expression indicated that the cells had not become senescent in any of the conditions [56], which is important as cellular senescence has been mechanistically linked to impaired angiogenesis [57]. Despite similar expression levels of VEGF with increasing confluence, the PEDF/VEGF ratio has been reported to increase with increasing confluence [58]. As PEDF is reported to be the most potent natural angiogenesis inhibitor [58], higher confluence may compromise pro-angiogenic properties and may explain the observed reduced efficacy of EVs isolated from highly confluent day 6 cultures.

Regarding changes in EV protein content, the measured reduction in HGF and FGF-2 was consistent with prior studies completed in our lab [28]. Though there was concern with the significant reduction of HGF levels, it did not appear to affect the functionality of these EV populations. While HGF is recognized as both an angiogenic and survival factor for endothelial cells, it has also been reported that it may participate in atherogenesis [59], which is a tissue response to injury involving chronic inflammation and the formation of fatty plaques within the arterial wall. If this is indeed the case, higher levels of HGF in EVs may not be seen as beneficial for the treatment of cerebrovascular conditions; however, further testing is needed. Ang-2 is an antagonist to Ang-1, and the ratio of Ang-1 to Ang-2 is important in maintaining vascular homeostasis [60]. Therefore, the reduction in Ang-2 protein could lead to improved Ang-1 function in contributing to EC angiogenesis and reduced EC permeability [44]. Unfortunately, Ang-1 protein content was not measured in the current study. MSC differentiation is associated with a reduction in CD105 expression [61] and could explain the reduced CD105 concentration in EVs isolated from SSBs on day 6, despite the increase in EV particle counts. While MSCs have been demonstrated to retain their defining characteristics both in PPRF-msc6 growth medium [33] and in SSBs [20], their expression of stemness genes is reduced at high confluence [52]. 

It was confirmed that EV yield (EV/cell) increased with exposure to shear stress in dynamic conditions, consistent with previous studies [26,30,31]. Furthermore, EV yield did not change between days 4 and 6 in SSBs (whereas cell density increased), indicating that EV yield was not a function of cell density or time of harvest during this period. The increase in EV yield under dynamic conditions could have been due to an increase in intracellular calcium, which has been shown to increase MSC proliferation rates without compromising their defining characteristics [62], as well as to upregulate EV release by several cell types [63,64,65]. Under dynamic conditions, increases in intracellular calcium levels have been shown to be induced by fluid shear via the mechanosensitive calcium channel TRPV4 [66]. Specifically, Taylor et al. reported calcium-dependent production of EVs originating from the plasma membrane (i.e., microvesicles) [64]. 

We previously reported a reduction in the percentage of syntenin-1 expressing cells in SSBs [28], likely due to an increase in the microvesicle to exosome ratio under dynamic conditions as syntenin-1 is known to be a specific marker of exosomes [32]. A study by de Almeida Fuzeta et al. reported a higher abundance of CD81, CD63, and syntenin in MSC-derived EVs isolated from vertical wheel bioreactors [26], while syntenin expression in the MSCs themselves was higher under static conditions. However, de Almeida Fuzeta et al. utilized precipitation for EV isolation followed by Western blot quantification of the total protein, whereas the current study utilized ultracentrifugation followed by single-particle EV analysis. If an upregulation in syntenin expression in MSCs translates to upregulated expression per EV particle, this could result in an overall larger protein quantification even if the number of syntenin expressing particles is lower. This could, therefore, contribute to the differences between the present study and that of de Almeida Fuzeta and colleagues, and it outlines the need to standardize EV isolation and characterization protocols. Other contributing factors could include culture parameters such as the culture medium, shear rate, microcarrier density, cell seeding rate, or cell confluence.

Functionally, EVs isolated from dynamic conditions were able to induce the proliferation of CMECs regardless of when they were harvested (days 4, 5, or 6). The induction of CMEC proliferation by EV populations derived from static MSC cultures was not observed, consistent with previous studies within our laboratory [19]. EVs from both static and dynamic MSC cultures induced tube formation in CMECs, similar to what was seen previously [19]. Though there were no significant functional differences in EVs isolated from the different culture conditions, EVs isolated from SSBs on day 5 were the only population to consistently promote a significant increase in meshed area compared to the control at the 3 h time point, which suggests that this specific EV population is taken up at a faster rate. Notably, at the 3 h time point, the quantified measures of tube formation correlated with the concentration of CD105 in the EV fractions. 

In this study, EV populations were compared on a per-volume-of-CM basis and not a basis of EV quantity. This was an intentional decision undertaken to compare bioprocess parameters as opposed to individual EVs. All conditions were normalized to process inputs. Therefore, compared to conditions involving the addition of EVs from static MSC cultures, a greater quantity of EVs was added to CMECs from day 5 and day 6 dynamic conditions, and fewer EVs were added from day 4 dynamic conditions. However, a correlation was not seen between function and EV quantity. Indeed, the concentrations of angiogenic proteins within the EV fractions were lower at longer time points on a per-EV basis; thus, it is likely that individual EV functionality changes with increasing confluence or a change in the cell state.

Several limitations to this study should be noted. All experiments completed within this study utilized a single MSC donor. In generating a bioprocess to specifically produce MSC-EVs for the purpose of inducing angiogenesis, it will be necessary to select donors that produce the most therapeutically effective EV populations. Further, though UC is the most commonly used method for EV isolation [67], it may not be the most advantageous method to isolate EV populations going forward. UC results in a lower EV purity compared to other methods and the high forces they are subject to may result in damage. Isolated EV pellets contain aggregates of proteins, nucleic acids, and lipids secreted by the cells which co-isolate with EVs due to similarities in size/density and/or due to molecular interactions at the EV surface [68]. Increasing evidence suggests that EVs are physically and functionally associated with proteins and nucleic acids both internally and externally [69,70,71]; therefore, further understanding of EVs and how isolation methods affect their purity and function is needed in order to select an optimal isolation protocol. 

This study utilized the immortalized hCMEC/d3 cell line as a model of cerebral angiogenesis. Though the use of primary-or-stem-cell-derived CMECs could provide a better model system, the expression levels of hCMEC/d3 cells have been found to be consistent with those of primary CMECs, and this cell line is well characterized as it has been used extensively as a model of the BBB [72,73]. However, preclinical trials are needed to evaluate the efficacy of the produced EVs on a whole system level, as well as to determine optimal dosing and time of treatment. Finally, this study did not evaluate a specific mechanism of action of MSC-EVs. Further studies are needed to understand how MSC-EVs contribute to translational or transcriptional changes in CMECs, and to elicit their mechanism(s) of action. A significant amount of research has pointed to the benefits of MSC-EVs being associated with their encased regulatory RNA molecules [74]. Further study of the RNA content in EVs from MSCs cultured under different conditions could enable a better understanding of the impact of such culture conditions and, overall, would contribute to the development of bioprocesses aimed at producing the most therapeutically relevant MSC-EV populations. 

To conclude, this study demonstrated a scalable bioprocess for producing, under clinically relevant conditions, MSC-EVs that have potential to promote angiogenesis in the brain for the treatment of ischemic conditions. It was found that MSC-EVs can be produced in scalable, dynamic SSBs, and these EVs stimulate angiogenesis in CMECs to a greater extent than MSC-EVs produced in standard static culture. Cell confluence or time of harvest was found to alter EV properties and should therefore be considered when optimizing bioprocesses for EV production. This study serves as a strong reference for the future development of bioprocesses targeted at producing MSC-EVs as therapeutics, which considers scalability and oxygen level in a clinically translatable serum-free medium.

## 4. Materials and Methods

### 4.1. MSC Culture

Human-adipose-derived MSCs (University of Calgary Health Research Ethics Board; ID: REB15-1005; female, age 24, BMI within normal range, abdominoplasty performed by a surgeon at the Foothills Hospital in Calgary, AB, Canada) were isolated from abdominal subcutaneous adipose tissue and characterized as described previously [75]. MSCs were expanded in serum-free PPRF-msc6 growth medium [33] as previously described [19,28]. Briefly, serial expansion was performed in normoxic (18.4% O_2_) conditions. At each passage, cells were inoculated at 5000 cells/cm^2^ into 12 mL of PPRF-msc6 in T-75 flasks and passaged every 72 h. For experiments, MSCs at passage 5 were inoculated at 5000 cells/cm^2^ in either static T-75 flasks (Thermo Fisher Scientific, Waltham, MA, USA) or in 100 mL SSBs (NDS Technologies, Vineland, NJ, USA) operated at 40 rpm (max shear stress of 1.6 dyn/cm^2^) [28] inside chambers maintained at 3% O_2_, 5% CO_2_, 37 °C, and 100% humidity in the headspace. The O_2_ level was maintained using a SubChamber system (BioSpherix, Parish, NY, USA) by displacing O_2_ with N_2_. N_2_ injection was regulated by a ProOx Model 110 oxygen controller (BioSpherix). Cytodex 3 microcarriers (2.3 g/L) were added to the SSBs to enable MSC attachment and were prepared as described previously [28]. Microcarriers were added to SSBs at a density matching that of the surface area per culture volume ratio of T-flasks (6.25 cm^2^/mL) to set a basis for experiments. 

For EV collection, the growth medium in each culture vessel was replaced with an equal volume of EV collection medium. EV collection medium was prepared by ultracentrifuging fresh PPRF-msc6 at 105,000 g for 18.5 h (Beckman Coulter Optima L-100K, 70 Ti rotor, 38,000 rpm, k factor = 148) and filtering (0.22 µm) the supernatant. This step was essential to remove proteins in the medium that typically co-isolate with EV fractions during the isolation process. The cells were washed twice in DPBS prior to the addition of EV collection medium. After a further 48 h, the EV collection medium was harvested from each vessel, and subsequently processed to isolate the EVs. For the static T-flask cultures, medium was replaced at day 2 and collected after 48 h on day 4. EV collection was carried out on days 2–4 as prior studies completed in our lab found this time period to yield the highest attainable concentrations of EVs and angiogenic proteins without affecting cell viability, growth, and MSC-specific surface marker expression [76]. For the SSBs, 3 time points were compared for collection of EVs: day 4, day 5, and day 6. For those collected on day 4, medium was replaced 48 h prior on day 2 (SSBs set 1). For those collected on day 5, medium was replaced on day 3 (SSBs set 1). For those collected day 6, medium was replaced both on day 2 and 4 (same SSBs as set 1).

### 4.2. RT-qPCR Analysis of MSCs

MSCs were washed twice with DPBS, lysed in Trizol reagent, and frozen at −20 °C. Total RNA was isolated and reverse transcribed as described previously [28]. Briefly, any potential contaminating DNA was digested using a RNase-Free DNase Set (Qiagen, Hilden, Germany) and RNA was isolated using an RNEasy Mini Kit (Qiagen). RT-qPCR was executed using an iCycler (Bio-Rad Laboratories Inc., Mississauga, ON, Canada) with human-specific primers for genes associated with angiogenesis (ANG1, FGF2, SDF1, and VEGF), proliferation (FGF2), anti-apoptosis (BCL2), oxidative stress (HIF1, HMOX1), and immunomodulation (IL-6, MCP1, TGFB1), as previously validated and outlined in Table 1 [19]. A total of 2 µg of RNA/sample was reverse transcribed using an Omniscript RT Kit (Qiagen), with template cDNA amplified using iQ SYBR Green Supermix (Bio-Rad Laboratories Inc.) as per the manufacturer’s reaction mix preparation and 2-step slow thermal cycling protocol. Annealing temperatures were optimized for each set of primers, and a melt curve analysis (55–95 °C, 0.5 °C increments for 0.05 min) was conducted for each primer pair to confirm amplicon specificity. Resultant data were analyzed using the delta-delta CT method, normalized to 18S.

### 4.3. EV Isolation

**Figure 6:**
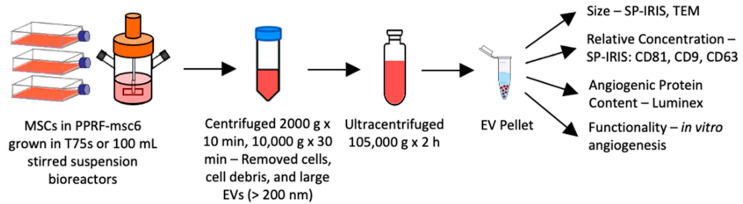
Isolation and characterization protocols for MSC-EVs.

Differential ultracentrifugation was used to isolate the EV fractions, as previously reported [19] and outlined in Figure 6. Briefly, expended medium was centrifuged at 2000 *g* and 4 °C for 10 min and then 10,000 *g* and 4 °C for 30 min to remove pelleted cell debris and large EVs (EVs < 200 nm remain in the supernatant). The supernatant was diluted with DPBS and ultracentrifuged at 105,000 *g* and 4 °C for 2 h (Beckman Coulter Optima L-100K, 70 Ti rotor, 38,000 rpm, k factor = 148). The pellet containing the EV fraction was re-suspended in (i) endothelial cell medium for use in functional in vitro experiments; (ii) DPBS for transmission electron microscopy (TEM) and single-particle interferometric reflectance imaging sensor (SP-IRIS) analyses; or (iii) RIPA buffer (1× with 10 µL/mL protease inhibitors (EMD Millipore, Burlington, MA, USA)) for biomolecular analyses. Resuspended EV fractions not used immediately were frozen at −80 °C for subsequent analyses. 

### 4.4. TEM

TEM was used to confirm the morphology and size of EVs using a Hitachi H7650 120 kV microscope (Hitachi High-Tech, Tokyo, Japan). Briefly, EVs were adsorbed to formvar coated copper mesh grids (Electron Microscopy Sciences, Hatfield, PA, USA) for 30 min, fixed in 2.5% glutaraldehyde for 15 min, washed twice with dH_2_O, stained with 2.6% uranyl acetate, washed twice again with dH_2_O, and dried at room temperature before being imaged at 80 kV.

### 4.5. SP-IRIS

An ExoView R100 (Nanoview, Boston, MA, USA) SP-IRIS device was used for particle counts, size, and concentration using EV-specific markers CD81, CD63, and CD9 and syntenin-1, as well as the negative marker GRP94. A total of 50 µL of each sample was incubated at room temperature overnight on a microarray tetraspanin chip. For particle concentration analyses, the chips were washed and stained with fluorescently conjugated antibodies CD81, CD63, and CD9. For cargo analyses, the chips were washed, and the captured particles were fixed, permeabilized, and then stained with fluorescently conjugated antibodies CD63, CD9, and syntenin-1 or CD63, CD9, and GRP94. The percentage of syntenin-1 and GRP94 was calculated by dividing by the total CD63 particle counts subject to the same washing and permeabilization steps.

### 4.6. Luminex Multiplex Analysis

Protein quantification was performed on lysed EV fractions using a Luminex-based Human Angiogenesis and Growth Factor 17-Plex Discovery Assay (Eve Technologies, Calgary, AB, Canada). This assay measured the concentration of the following biomarkers: Ang-2; BMP-9; EGF; CD105; endothelin-1; FGF-1; FGF-2; follistatin; G-CSF; HB-EGF; HGF; IL-8, leptin; PLGF; VEGF-A; VEGF-C; VEGF-D. Eve Technologies utilizes a Bio-Plex 200 device, which detects fluorescent conjugates bound to analyte-specific beads and quantifies results according to a standard curve. BMP-9, EGF, endothelin-1, follistatin, HB-EGF, leptin, and VEGF-D concentrations were under the detection limit and were therefore not included in the analysis. 

### 4.7. CMEC Culture

The immortalized human CMEC line (hCMEC/D3) (Cedarlane, Burlington, ON, Canada) was chosen as an in vitro model of the BBB as it is widely used and amendable in studying endothelial cell mechanisms with relevance to the brain [72]. CMECs were inoculated into tissue culture flasks and expanded in endothelial growth medium (EGM-2) (Lonza, Basel, Switzerland). EGM-2 was prepared by supplementing endothelial basal medium (EBM-2) with EGM-2 SingleQuot supplements, which consist of serum, hormones, and growth factors (proliferative and/or angiogenic) that stimulate endothelial cell growth. Medium changes were performed every 2 days, and cells were passaged at 90% confluence.

### 4.8. Proliferation Assay

CMECs were inoculated into 96-well plates at 5000 cells/cm^2^ in 100 µL of EGM-2 for 12 h. After 12 h, the EGM-2 was removed and the medium was replaced with either basal medium (EBM-2) alone (negative control), growth medium (EGM-2) alone (positive control), or with EBM-2 or EGM-2 supplemented with EVs produced by MSCs cultured in static T-flasks or dynamic SSBs. EVs were added to reach a concentration equivalent to 20× the CM from which they were isolated (i.e., 1 mL of CMEC culture medium would contain EVs isolated from 20 mL of MSC conditioned medium). This dose was used as it was previously shown to be stimulatory for these cells [19]. EV populations were compared on the basis of CM volume, which represents equivalent process inputs (i.e., equal volume of cells, surface area and medium on day 0). After 48 h, the cells were washed with DPBS and the well plates were frozen at −80 °C. The amount of DNA in each well was quantified using a CyQUANT Cell Proliferation Assay Kit (Invitrogen, Thermo Fisher Scientific) as per the manufacturer’s instructions. Briefly, the well plates were thawed to room temperature, and then 200 µL of CyQUANT GR/cell lysis buffer was added to each well and incubated at room temperature for 5 min in the dark. Sample fluorescence was read using a microplate reader at 485 nm excitation and 525 nm emission maxima. Optical density (OD) readings were converted to cell number using a standard curve of known DNA concentration and approximating DNA per cell at the weight of the human genome, 6.41 pg [77].

### 4.9. Tube Formation Assay

The 24-well plates were coated with 200 µL of Geltrex LDEV-Free Reduced Growth Factor Basement Membrane Matrix (ThermoFisher Scientific, Waltham, MA, USA) and incubated at 37 °C for a minimum of 30 min prior to use. CMECs were inoculated into the Geltrex-coated 24-well plates at 30,000 cells/cm^2^ in EBM-2 with or without the addition of EVs from each condition. EVs were added at a concentration of 20×. The final well volume for each condition was 0.5 mL. Tube formation was evaluated at 3, 6, 9, and 12 h and analyzed using ImageJ Angiogenesis Analyzer software [78]. Total meshed area, total segment length, and branching interval were used for analysis as they were considered the most stable parameters.

### 4.10. Statistical Methods

Data are presented as mean ± standard deviation (SD). One-way ANOVAs followed by post hoc analysis using the Bonferroni multiple comparisons test was used to compare between conditions. The difference in means was determined to be significant if *p* < 0.05. GraphPad Prism was utilized to compute all statistics.

## Figures and Tables

**Table 1:** Human-specific primers used for RT-qPCR (F: forward; R: reverse).

Gene	Primer Sequence (5′-3′)	Origin
*18S*	F: TGG TCG CTC GCT CCT CTC C R: CGC CTG CTG CCT TCC TTG G	NR_003286
*ANG1*	F: CCT GAT CTT ACA CGG TGC R: GCT TTC ATA ATC GCT TCT	NM_001314051
*BCL2*	F: GAT GAC TGA GTA CCT GAA CC R: AGT TCC ACA AAG GCA TCC	EU287875
*FGF2*	F: CGC GGT TGC AAC GGG AT R: GGG TTC ACG GAT GGT TGT CT	NM_27968
*HIF1*	F: CCA GTT ACG TTC CTT CGA TCA GT R: TTT GAG GAC TTG CGC TTT CA	NM_001243084
*HMOX1*	F: ATG ACA CCA AGG ACC AGA GC R: GTG TAA GGA CCC ATC GGA GA	NM_002133
*IL6*	F: TCA ATA TTA GAG TCT CAA CCC CCA R: TTC TCT TTC GTT CCC GGT GG	NM_000600
*MCP1*	F: GCA ATC AAT GCC CCA GTC AC R: TCT TTG GGA CAC TTG CTG CT	S71513
*SDF1*	F: GGA CTT TCC GCT AGA CCC AC R: GCC CGA TCC CAG ATC AAT GT	NM_199168
*TGFB1*	F: GGG GAA ATT GAG GGC TTT CG R: CCA GGA CCT TGC TGT ACT GC	NM_000660
*VEGF*	F: ACG GTC CCT CTT GGA ATT GG R: GGC CGC GGT GTG TCT A	M32977

## Data Availability

The original contributions presented in the study are included in the article/Supplementary Material. Further inquiries can be directed to the corresponding author.

## Supplementary Materials

The following supporting information can be downloaded at: https://www.mdpi.com/article/10.3390/ijms25105219/s1.
