# Supplementary material for: Extracellular Vesicles Generated by Mesenchymal Stem Cells in Stirred Suspension Bioreactors Promote Angiogenesis in Human-Brain-Derived Endothelial Cells
Source: Int J Mol Sci. 2024 May 10;25(10):5219. doi: 10.3390/ijms25105219 (PMC11121007; doi:10.3390/ijms25105219)
Supplement: Supplementary file 1 [file ijms-25-05219-s001.zip › ijms-2971803-supplementary.pdf]

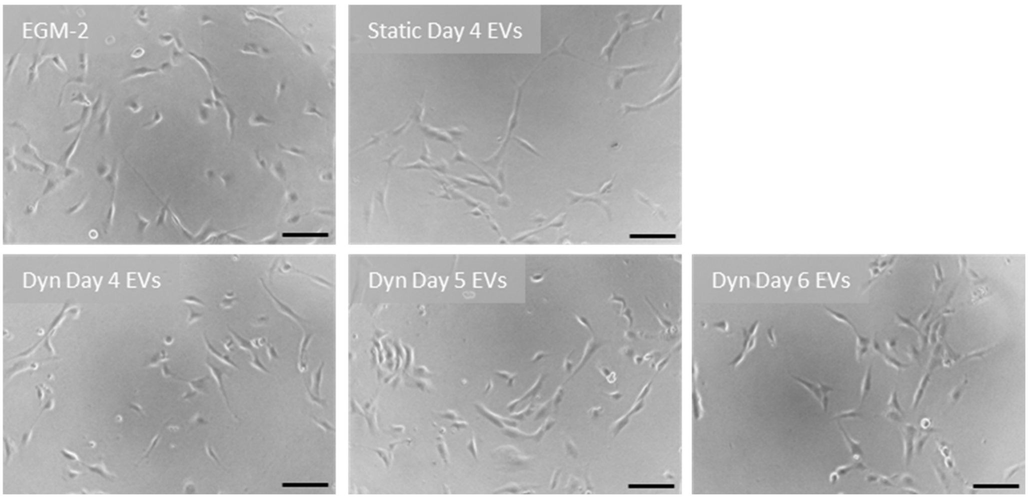

Figure S1. Photomicrographs of CMECs 48 h following treatments with EVs resuspended in EGM-2. Scale bar = 100 μm. Abbreviations: CMEC, cerebral microvascular endothelial cell; EBM, endothelial basal medium; EGM, endothelial growth medium; EV, extracellular vesicle; MSC, mesenchymal stem cell.

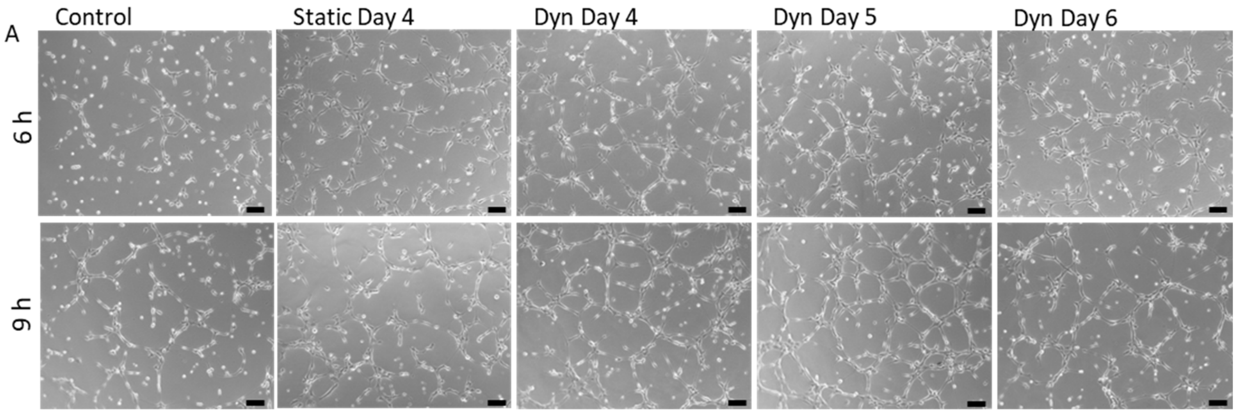

Figure S2. Phase contrast images of CMEC tubes formed after 6 and 9 h on a Geltrex matrix exposed to EBM-2 (control), or EBM-2 with the addition of EVs isolated from static MSC cultures on day 4, or SSB (dyn) MSC culture on day 4, 5, or 6 (scale bar = 100 μm). Abbreviations: CMEC, cerebral microvascular endothelial cell; EBM, endothelial basal medium; EV, extracellular vesicle; MSC, mesenchymal stem cell.
